# Acinetobacter pengchengensis sp. nov., isolated from the urban wastewater of Shenzhen, Guangdong Province, China

**DOI:** 10.1099/ijsem.0.007174

**Published:** 2026-06-17

**Authors:** Panpan Yang, Bingchan Guo, Yinghui Li, Dingjie Huang, Xi Yang, Lei Wang, Ziqi Wu, Yanpeng Cheng, Qinghua Hu

**Affiliations:** 1 School of Public Health, Shanxi Medical University, Taiyuan 030000, PR China; 2 Shenzhen Center for Disease Control and Prevention, Shenzhen 518000, PR China

**Keywords:** *Acinetobacter*, new species, taxonomic studies

## Abstract

Two bacterial strains, designated LH3_13ᵀ and NS4_7, were isolated from urban wastewater samples collected in Shenzhen, China. Cells were aerobic, Gram-stain-negative, oxidase-negative, catalase-positive, non-motile and short rods to coccobacilli. Phylogenetic analysis based on 16S rRNA gene sequences placed the two strains within the genus *Acinetobacter*, showing the highest sequence similarity to *‘Acinetobacter seohaensis’* SW-100 (98.44–98.39%). Among the validly published species, the closest relatives were *Acinetobacter indicus* and *Acinetobacter baumannii*, with sequence similarities of 97.5–97.4% and 96.5–96.3%, respectively, values below the generally accepted species demarcation threshold. Whole-genome sequencing and comparative genomic analyses further supported the novelty of the strains. The average nucleotide identity (ANI) values between strains LH3_13ᵀ and NS4_7 and their closest related species, *Acinetobacter towneri* DSM 14962ᵀ, were 92.5–92.6%, and the corresponding digital DNA–DNA hybridization (dDDH) values were 50.2–50.6%, both clearly below the species delineation thresholds. In contrast, ANI and dDDH values between LH3_13ᵀ and NS4_7 were 97.6 and 82.7%, respectively, indicating that they belong to the same species. Core-genome phylogenomic analysis based on 81 bacterial core genes confirmed that the 2 strains formed a distinct and well-supported clade within the genus *Acinetobacter*. Phenotypic, physiological and chemotaxonomic characteristics further differentiated strains LH3_13ᵀ and NS4_7 from their closest phylogenetic relatives. Based on the combined evidence from phylogenetic, genomic, phenotypic and chemotaxonomic analyses, strains LH3_13ᵀ and NS4_7 represent a novel species of the genus *Acinetobacter,* for which the name *Acinetobacter pengchengensis* sp. nov. is proposed. The type strain is LH3_13ᵀ (=JCM 37796ᵀ=GDMCC 1.4974ᵀ).

## Introduction

The genus *Acinetobacter* was formally proposed by Brisou and Prevot in 1954 [1], with *Acinetobacter calcoaceticus* designated as the type species. It is currently placed within the family *Moraxellaceae* [2]. According to the List of Prokaryotic names with Standing in Nomenclature (LPSN; accessed in October 2025), the genus currently comprises 87 species with validly published names, including the taxonomically and clinically significant species *A. calcoaceticus*, *Acinetobacter lwoffii* and *Acinetobacter baumannii*. Members of this genus were isolated from environmental samples (forest ecosystems [3], water [4,7], soil [8,11], clinical specimens [12,15] and animal samples (faeces [16] and organs [17,18]). They are Gram-negative, non-motile and coccobacillary bacteria, with Q9 as the predominant quinone and C_18 : 1_* ω9*c as the major fatty acid. Numerous studies have revealed that *Acinetobacter* species pose a significant threat to clinical infections, with *A. baumannii* in particular [19] being a focus of research due to its multidrug resistance [14,20]. Moreover, species in the genus *Acinetobacter* exhibit strong environmental adaptability and have attracted considerable attention because of their potential roles in environmental remediation [21] and biodegradation [22]. In our recent studies [23,25], we found that viral signals in urban wastewater closely tracked population-level dynamics of infectious diseases, highlighting wastewater as a highly human-relevant microbial interface. Based on these findings, we initiated a systematic effort to isolate and characterize bacterial taxa from wastewater in order to better understand their diversity and taxonomic placement within established lineages. This work also contributes to the development of a pathogen identification platform for the future early detection of emerging bacterial infectious diseases. We isolated bacteria from urban wastewater and recovered two strains belonging to the genus *Acinetobacter*. In this study, we have characterized these two isolates (LH3_13^T^ and NS4_7) using a polyphasic taxonomic approach and propose that they represent a novel species within the genus *Acinetobacter*.

## Methods

### Isolation and culture of bacterial strains

Urban wastewater samples were collected on 25 June 2024 from the Longhua District Phase II Water Purification Plant (22° 41′ 4″ N/114° 3′ 7″ E) and the Nanshan District Water Purification Plant (22° 31′ 8″ N/113° 54′ 21″ E) in Shenzhen, Guangdong Province, China. The wastewater samples were subjected to a rigorous resuspension, homogenization, centrifugation and washing with sterile saline. The resulting sediment was collected, transferred to 2-ml tubes and centrifuged again to completely remove the supernatant, and the retaining pellet was stored at −80 °C. After thawing, the wastewater sediment samples were each diluted with 1 ml of 0.85% sterile saline to create solution samples, and 100 µl of each was further diluted 100-fold. These diluted samples were then spread on brain heart infusion (BHI) and Reasoner’s 2A agar (R2A) plates and incubated aerobically at 15–40 °C for 2–3 days. A total of 383 isolates were recovered under these culture conditions. Among them, two greyish-white colonies, designated LH3_13^T^ and NS4_7, were initially isolated on BHI agar after aerobic incubation at 28 °C, selected based on colony morphology, purified and subjected to further analyses. Strain LH3_13^T^ was isolated from the Longhua District Phase II Water Purification Plant, whereas strain NS4_7 was isolated from the Nanshan District Water Purification Plant. The type strain LH3_13^T^ has been deposited in the Guangdong Microbial Culture Collection Center (GDMCC) and the Japan Collection of Microorganisms (JCM), with accession numbers GDMCC 1.4974^T^ and JCM 37796^T^, respectively.

### 16S rRNA gene sequencing and phylogenetic analysis

The nearly full-length 16S rRNA gene sequences of strains LH3_13^T^ and NS4_7 were amplified and sequenced using the universal primers 27F (5′-AGA GTT TGA TCC TGG CTC AG-3′) and 1492R (5′-ACG GCT ACC TTG TTA CGA CTT-3′) [26]. The obtained 16S rRNA gene sequences were subjected to blast analysis against the National Center for Biotechnology Information (NCBI) database (https://www.ncbi.nlm.nih.gov/) and the EzBioCloud website (https://www.ezbiocloud.net) for preliminary taxonomic assignment.

To further infer the phylogenetic relationships of the isolates, 16S rRNA gene sequences of all type strains of the genus *Acinetobacter* were retrieved from the LPSN database and aligned using mega X [27]. Phylogenetic trees were reconstructed using the neighbour-joining (NJ), maximum-likelihood (ML) and maximum parsimony (MP) algorithms with 1,000 bootstrap replications, and evolutionary distances were calculated based on the Kimura two-parameter model [28].

### Genome sequencing, assembly and genome-based analyses

Genomic DNA was extracted using the BayBiopure Magnetic Bead-Based Bacterial Genomic DNA Extraction Kit. In the initial stage of the study, draft genome sequences of strains LH3_13^T^ and NS4_7 were generated using the Illumina HiSeq PE150 platform and assembled from short-read data with SOAPdenovo [29]. Subsequently, to improve genome assembly quality, the two strains were further sequenced using Oxford Nanopore long-read technology. The Nanopore long reads were combined with the previously generated Illumina short reads for hybrid assembly using Unicycler v0.4.7 [30], yielding final hybrid genome assemblies for both strains. Genome completeness and contamination were assessed using CheckM2.

Genomic relatedness was evaluated based on average nucleotide identity (ANI) and digital DNA–DNA hybridization (dDDH). ANI values were calculated using the OrthoANIu algorithm [31], and dDDH values were computed using the GGDC platform [32]. To further resolve the phylogenetic placement of strains LH3_13^T^ and NS4_7, a revised genome-scale phylogenetic analysis was performed using the UBCG2 pipeline [33] based on a curated set of bacterial core genes. The analysis included 95 genomes, comprising the two isolates, 91 publicly available *Acinetobacter* genomes, ‘*Acinetobacter seohaensis*’ as an additional closely related reference, and *Psychrobacter immobilis* DSM 7229^T^ as the outgroup. In total, 81 bacterial core genes were extracted, individually aligned and concatenated into a supermatrix of 23,821 amino acid positions. Gap-rich columns were filtered using the default UBCGtree setting. The phylogenomic tree was inferred using IQ-TREE v2.0.3 [34] under an ML framework, with the best-fit model selected by ModelFinder (LG+F+R8). Branch support was assessed using SH-aLRT with 1,000 replicates and ultrafast bootstrap with 1,000 replicates, with branch-length optimization by nearest-neighbour interchange. The tree was visualized using Dendroscope 3 [35].

### Functional inference based on genome sequence

Amino acid sequences predicted from the genomes of strains LH3_13^T^, NS4_7, *Acinetobacter towneri* DSM 14962^T^ and *A. calcoaceticus* NCTC 12983ᵀ (=ATCC 23055ᵀ) [36] were submitted to the BlastKOALA platform for functional annotation based on Kyoto Encyclopedia of Genes and Genomes (KEGG) orthology groups [37]. Gene annotation was performed using the software’s default parameters, and the results are presented numerically, representing the number of genes successfully annotated to specific functional categories.

Functional gene annotation was performed against the Virulence Factor Database (VFDB) [38] and the Comprehensive Antibiotic Resistance Database (CARD) [39]. Putative virulence-associated homologues were identified for strains LH3_13^T^ and NS4_7 using the online VFanalyzer pipeline in VFDB, with genome assemblies uploaded in FASTA format under the private genome analysis mode. For draft/complete genome analyses, candidate matches were retained based on the best hit using the default VFDB thresholds of >90% sequence identity and >80% coverage. Because the online VFanalyzer output reports annotated homologous loci but does not explicitly provide per-hit identity and coverage values for each predicted match, the VFDB results are presented here according to the default screening criteria of the platform rather than as gene-by-gene percentage values. All VFDB-derived annotations were interpreted conservatively as genomic homologues to previously reported virulence-associated genes, without implying confirmed biological function, pathogenicity or ecological role in the studied strains. For CARD-based analysis, genome assemblies of strains LH3_13^T^ and NS4_7 were analysed using the Resistance Gene Identifier with FASTA sequences submitted in DNA sequence mode. The analysis was performed using the ‘Perfect and Strict hits only’ criterion under the high-quality/coverage setting. CARD-derived matches were interpreted conservatively as database-annotated genomic homologues to previously described antimicrobial resistance-associated genes, without implying confirmed phenotypic resistance in the studied strains.

### Phenotypic, physiological and biochemical analyses

Based on 16S rRNA similarity, genomic relatedness and the phylogenetic positions of the two isolates, *A. towneri* CCUG 50769^T^ (=DSM 14962^T^) was acquired from the Culture Collection University of Gothenburg (CCUG) for subsequent identification studies.

For morphological observation, strains LH3_13^T^ and NS4_7 were cultivated on BHI agar at 28 °C for 2 days. Cell morphology was examined under a light microscope, and Gram staining was performed using a commercially available Gram-staining kit. In addition, morphological features were further observed by scanning electron microscopy using cells harvested at the early logarithmic and stationary phases under optimal growth conditions.

For cultural characterization, both strains were inoculated onto eight different media, including Columbia blood agar, R2A, BHI agar, nutrient agar, tryptic soy agar, Luria–Bertani (LB) agar, MacConkey agar and Mueller–Hinton agar, and incubated at 28 °C for 2–3 days. The effect of aerobic and anaerobic conditions on growth was assessed on BHI agar after incubation for 2–4 days. Subsequently, BHI broth was used as the basal medium to determine the effects of different temperatures, pH values and NaCl concentrations on growth. Growth at different temperatures was tested at 4, 10, 20, 30, 35, 37, 40 and 42 °C. The pH range for growth was determined in BHI broth adjusted to pH 3.0–11.0 at intervals of 1.0 pH unit. Salt tolerance was assessed in BHI broth supplemented with 0, 0.5, 1.0, 2.0, 3.0, 4.0, 5.0, 6.0, 7.0 and 8.0% (w/v) NaCl. Growth under each condition was monitored by measuring the optical density at 600 nm (OD_600_) at 2 h intervals using a spectrophotometer.

To further characterize the physiological and biochemical properties of the novel strains and the reference strain, API ZYM and API 20NE strips were used according to the manufacturers’ instructions. Catalase activity was determined using the ID Colour Catalase kit, and oxidase activity was tested using API reagents.

### Chemotaxonomic analyses

Fatty acid methyl esters (FAMEs) were extracted and analysed using the Sherlock Microbial Identification System (MIDI) according to the manufacturer’s standard protocol, which includes saponification, methylation, extraction and washing steps, followed by gas chromatographic analysis [40]. The fatty acid profiles of strains LH3_13^T^ and NS4_7, and the reference strain *A. towneri* DSM 14962^T^ were determined in parallel under the same experimental conditions. Respiratory quinones in strain LH3_13^T^ and polar lipids were analysed according to standard procedures, using reverse-phase HPLC and two-dimensional (2D)-TLC, respectively [41].

The peptidoglycan structure of strain LH3_13^T^ was determined according to standard procedures [42]. In brief, purified cell walls were obtained by mechanical disruption of cells followed by sequential washing with SDS and saline. The peptidoglycan was hydrolysed with 6 M HCl at 100℃ for 16 h. The hydrolysate was dried, dissolved in distilled water and analysed by 2D-TLC on cellulose plates. The configuration of diaminopimelic acid (DAP) was specifically confirmed using an appropriate standard to distinguish between the meso- and ll-isomers. Amino acids were visualized by spraying with the ninhydrin reagent. The identities and molar ratios of the amino acids were determined by comparison with commercial standards, including alanine (Ala), glutamic acid (Glu), glycine (Gly), aspartic acid (Asp), lysine (Lys), ornithine (Orn) and meso-DAP. Whole-cell sugars were analysed by TLC using appropriate sugar standards [42].

### Accession numbers

The nearly full-length 16S rRNA gene sequences of strains LH3_13^T^ and NS4_7 have been deposited in GenBank under accession numbers PQ035156 and PQ035159, respectively. Early draft whole-genome shotgun assemblies based on Illumina sequencing were deposited in GenBank under accession numbers JBOWQO000000000 and JBQUYF000000000 for strains LH3_13^T^ and NS4_7, respectively. The final hybrid genome assemblies of strains LH3_13^T^ and NS4_7, generated from combined Oxford Nanopore and Illumina sequencing data, have been deposited in the European Nucleotide Archive (ENA) under accession numbers ERZ29227411 and ERZ29227412, respectively.

## Results and discussion

### 16S rRNA gene-based phylogenetic analysis

**Fig. 1.**
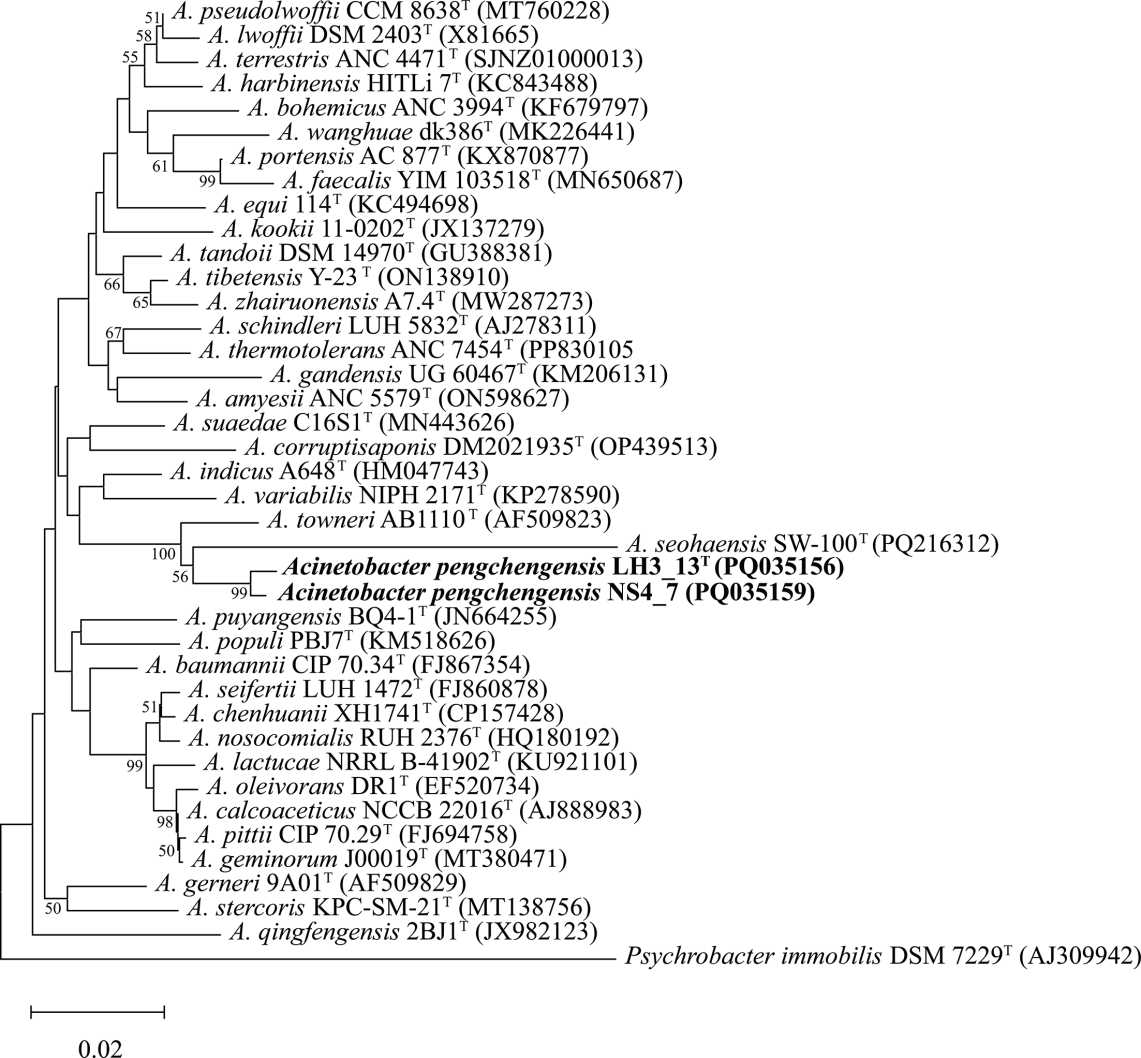
NJ tree based on the 16S rRNA gene. The tree shows the taxonomic position of strains LH3_13^T^, NS4_7 and other closely related species, including ‘*A. seohaensis*’ SW-100. The numerals (values >50% are noted) indicate the percentage of bootstrap samplings as derived from 1,000 replications. The sequence of *P. immobilis* DSM 7229ᵀ serves as an outgroup. Bar, 0.02 substitutions per nucleotide position.

The 16S rRNA gene sequence identity analysis revealed that the similarity between strain LH3_13^T^ and NS4_7 was 99.9%. Based on the NCBI blast results, the highest-scoring hit for both strains was ‘*A. seohaensis*’ SW-100, with 16S rRNA gene sequence similarities of 98.44% for strain LH3_13^T^ and 98.39% for strain NS4_7. Among the validly published species, the closest blast matches were *Acinetobacter indicus*, including the type strain A648^T^, which showed sequence similarities of 97.51% for strain LH3_13^T^ and 97.52% for strain NS4_7. Other closely related validly published species included *A. baumannii* JCM 6841^T^, showing sequence similarities of 96.46% for strain LH3_13^T^ and 96.29% for strain NS4_7. These values were all below the 98.7% threshold generally recommended for species delineation [43,44], suggesting that the two strains may represent a novel species. As shown in Figs 1, S1 and S2 (available in the online Supplementary Material), the topological structures of the phylogenetic trees constructed based on the 16S rRNA gene were consistent, indicating that strains LH3_13^T^ and NS4_7 formed a distinct lineage most closely related to *A. towneri* DSM 14962^T^. In addition, ‘*A. seohaensis*’ SW-100, currently listed in LPSN as a heterotypic synonym of *A. towneri*, was placed in the same lineage as *A. towneri*, consistent with the later genome report on DSM 16313 [45].

### Genome characteristics

Whole-genome sequencing followed by hybrid assembly generated genomes of 2,827,812 bp for strain LH3_13ᵀ and 3,061,065 bp for strain NS4_7, distributed across 3 and 6 contigs, respectively. The corresponding N50 values were 2,797,954 bp and 2,772,018 bp. The DNA G+C contents of both genomes were 41.1 mol%, which is within the range reported for members of the genus *Acinetobacter* [3]. Genome annotation predicted 2,758 genes in LH3_13ᵀ and 2,980 genes in NS4_7, including 2,659 and 2,879 protein-coding genes, 21 rRNA genes in each strain, 77 and 79 tRNA genes and 1 tmRNA gene in each genome. Genome quality assessment using CheckM2 indicated completeness values of 100.0% for both genomes and contamination values of 0.01% and 0.07% for LH3_13ᵀ and NS4_7, respectively (Table S1).

**Fig. 2.**
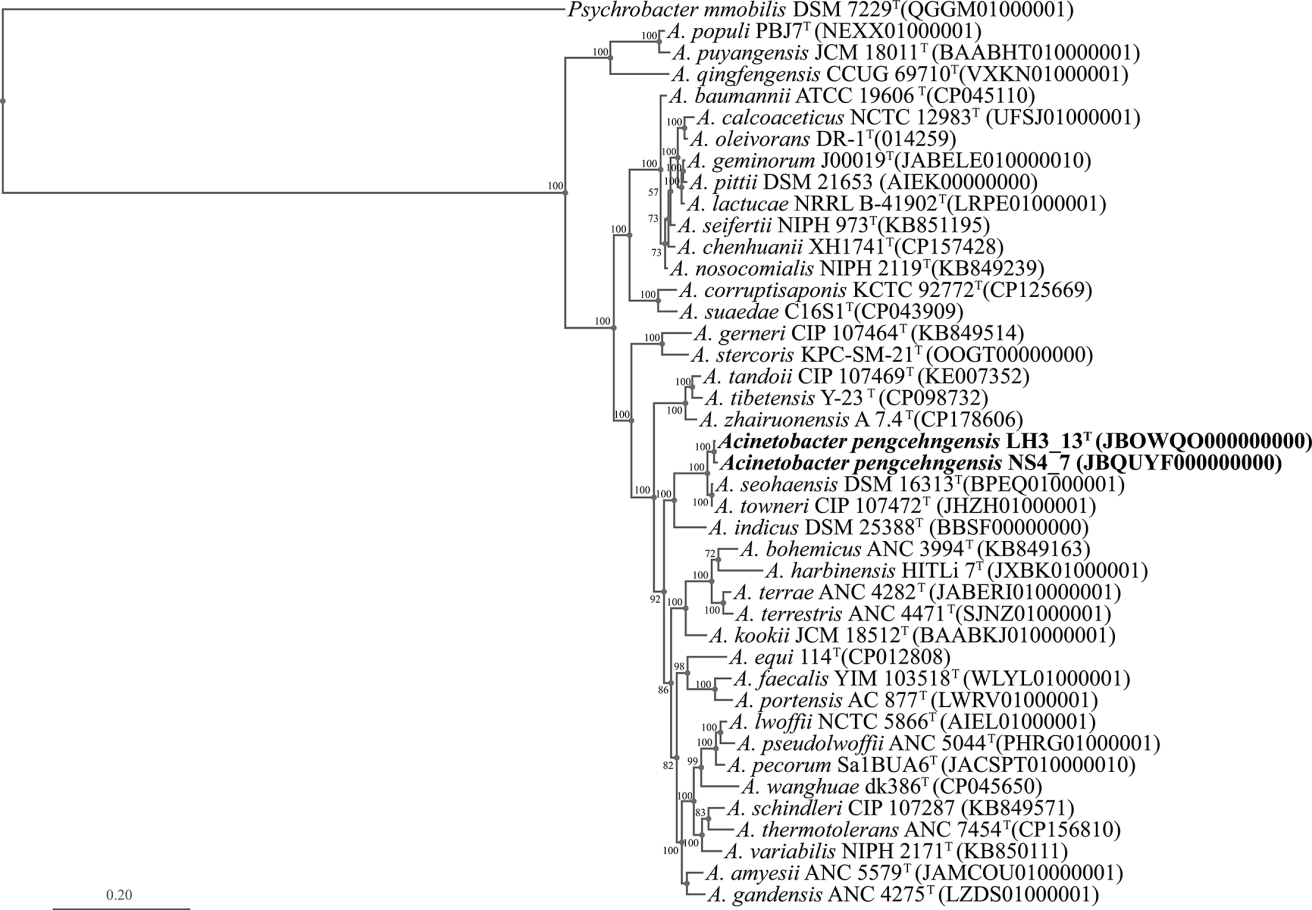
Phylogenomic tree of *Acinetobacter* strains LH3_13^T^, NS4_7 and closely related species. This tree is constructed based on 81 bacterial core genes from 95 genome sequences. The numbers in the tree indicate ultrafast bootstrap support values (%) calculated from 1,000 replicates. The sequence of *P. immobilis* DSM 7229ᵀ serves as an outgroup. Bar, 0.20 substitutions per nucleotide position.

As detailed in Table 1, the ANI value between strains LH3_13ᵀ and NS4_7 was 97.6%, indicating that they belong to the same species. The corresponding dDDH value was 82.7%, exceeding the 70% threshold for species delineation. In contrast, ANI values between LH3_13^T^ or NS4_7 and their closest related type strain, *A. towneri* DSM 14962ᵀ, were 92.6% and 92.5%, respectively, and below the species cutoff of 95–96% [46]. Their dDDH values relative to *A. towneri* were 50.2% and 50.6%, also below the 70% threshold [47]. Comparisons with other type strains yielded even lower ANI (76.7–78.5%) and dDDH (21.6–22.6%) values. Together, these genomic indices support the assignment of strains LH3_13ᵀ and NS4_7 to the same species and clearly distinguish them from recognized species of the genus *Acinetobacter*. A phylogenomic tree based on 81 bacterial core genes from 95 genomes (Fig. 2) showed that strains LH3_13ᵀ and NS4_7 formed a distinct lineage within the genus *Acinetobacter*. In the resulting tree, the two strains clustered most closely with *A. towneri* DSM 14962ᵀ. In addition, ‘*A. seohaensis*’ SW-100, which is listed in LPSN as not validly published and as a synonym (and no standing) of *A. towneri*, was placed in the same lineage as *A. towneri*, consistent with the later genome report on DSM 16313 [45].

**Table 1.** ANI/dDDH between *Acinetobacter pengchengensis* strains and the type strains of closely related species

ANI/dDDH	LH3_13^T^	NS4_7	1	2	3
LH3_13^T^	–	97.6/82.7	92.6/50.2	76.8/21.8	77.7/22.1
NS4_7	97.6/82.7	–	92.5/50.6	76.7/21.8	78.5/22.6
1	92.6/50.2	92.6/50.6	–	76.7/21.6	76.9/21.7
2	76.9/21.8	76.7/21.8	76.6/21.6	–	90.8/43.1
3	76.7/22.1	77.0/22.6	76.5/21.7	90.5/43.1	–

1, *A. towneri* DSM 14962T=CIP 107472T; 2, *Acinetobacter tibetensis* Y-23T; 3, *Acinetobacter tandoii* DSM 14970T=CIP 107469T.

### Functional inference analysis

The distribution of genes within KEGG functional categories revealed similarities between the two newly isolated strains and the closely related species *A. towneri* DSM 14962^T^ in several metabolic groups, including global overview maps, energy metabolism, amino acid metabolism, xenobiotics biodegradation and metabolism and cellular community-related pathways (Table 2). However, strains LH3_13^T^ and NS4_7 harboured a higher number of predicted genes associated with nucleotide metabolism, cofactor biosynthesis, nitrogen metabolism and nitrogen cycle-related functions, which were less represented in *A. towneri* DSM 14962^T^. Notably, nitrogen cycle-related genes were identified in the two isolates but were absent in their closest relative. Conversely, genes associated with benzoate degradation and several other aromatic compound degradation pathways were less represented in LH3_13ᵀ and NS4_7 than in *A. towneri* DSM 14962^T^. Furthermore, comparisons with the type species strain *A. calcoaceticus* NCTC 12983^T^ (=ATCC 23055^T^) revealed that, despite some quantitative differences in pathway annotation, the four strains generally exhibited broadly similar patterns across these functional categories. These comparative results indicate overall intrageneric similarity between the isolates and their close relatives, while supporting species-level differentiation based on stable genome-derived functional differences.

**Table 2.** Predicted presence of genes in six major KEGG functional categories in strains LH3_13^T^ and NS4_7 and related *Acinetobacter* type strains

Pathway and module	1	2	3	4
**Pathway**				
**Global and overview maps**				
01100 Metabolic pathways	473	470	479	602
01110 Biosynthesis of secondary metabolites	214	212	212	259
01120 Microbial metabolism in diverse environments	134	133	151	219
01230 Biosynthesis of amino acids	94	93	93	97
01232 Nucleotide metabolism	31	32	30	33
01240 Biosynthesis of cofactors	107	106	103	112
01220 Degradation of aromatic compounds	12	12	30	30
01310 Nitrogen cycle	3	3	0	3
**Energy metabolism**				
00910 Nitrogen metabolism	11	11	6	11
00920 Sulphur metabolism	18	18	19	29
**Amino acid metabolism**				
00330 Arginine and proline metabolism	6	6	5	13
00340 Histidine metabolism	9	9	9	13
00350 Tyrosine metabolism	5	5	6	14
00360 Phenylalanine metabolism	4	4	10	28
**Xenobiotic biodegradation and metabolism**				
00362 Benzoate degradation	18	18	29	29
00627 Aminobenzoate degradation	2	2	2	9
00364 Fluorobenzoate degradation	6	6	7	7
00625 Chloroalkane and chloroalkene degradation	3	3	3	3
00361 Chlorocyclohexane and chlorobenzene degradation	2	2	10	3
00623 Toluene degradation	2	2	10	3
00622 Xylene degradation	4	4	7	8
00930 Caprolactam degradation	2	2	3	5
00626 Naphthalene degradation	3	3	3	4
00984 Steroid degradation	2	3	5	6
00621 Dioxin degradation	0	0	3	5
**Cellular community – prokaryotes**				
02024 Quorum sensing	22	22	22	28
05111 Biofilm formation – *Vibrio cholerae*	18	18	19	21
02025 Biofilm formation – *Pseudomonas aeruginosa*	15	14	24	25
02026 Biofilm formation – *Escherichia coli*	13	13	9	14

Strains: 1, LH3_13T; 2, NS4_7; 3, *A. towneri* DSM 14962T; 4, *A. calcoaceticus* NCTC 12983T (=ATCC 23055T).

VFDB-based annotation indicated that strains LH3_13^T^ and NS4_7 displayed largely overlapping profiles of virulence-associated homologues, including homologues related to surface polysaccharide-associated loci, catalase (*katA*) and capsule-associated loci. In particular, homologues of *pgaA*, *pgaB* and *pgaC*, which are associated with PNAG (poly-*β*-1,6-*N*-acetylglucosamine)-related polysaccharide pathways, were identified in both strains, whereas *pgaD* was not detected in either strain. Overall, these results indicate that LH3_13^T^ and NS4_7 possess broadly similar sets of VFDB-annotated genomic homologues.

Annotation using CARD identified a limited number of antimicrobial resistance-associated homologues in strains LH3_13^T^ and NS4_7. In strain LH3_13^T^, two perfect hits were detected, corresponding to *msrE* and *mphE*, whereas in strain NS4_7, one strict hit corresponding to *aadT* was identified. All three matches were assigned by the protein homologue model. The *msrE* and *mphE* hits in LH3_13^T^ each showed 100.0% identity across 100.0% of the reference sequence, whereas the *aadT* hit in NS4_7 showed 97.82% identity across 100.0% of the reference sequence.

These KEGG-, VFDB- and CARD-based annotations are interpreted here only as database-derived genomic homologues or predicted functional assignments and do not, by themselves, demonstrate corresponding phenotypic traits.

### Phenotypic, physiological and chemotaxonomic characteristics

The results showed that both bacteria were aerobic and could grow on the tested culture media. Consistent with the phenotypic characteristics of the genus *Acinetobacter*, strains LH3_13^T^ and NS4_7 grew on BHI agar, formed grayish-white colonies, were Gram-stain-negative and exhibited short rods to coccobacilli with cell sizes of ~0.7–1.5×1.0–2.5 µm (Fig. S3). Comparison with the closely related species *A. towneri* DSM 14962ᵀ and the type species *A. calcoaceticus* ATCC 23055ᵀ showed overall similar colony and cellular morphologies (Table 3), indicating intrageneric consistency in these characteristics. Furthermore, strains LH3_13^T^ and NS4_7 were strictly aerobic and grew at temperatures ranging from 10 to 42 ℃ (optimal at 30–35 ℃). Both strains tolerated 0–5% (w/v) NaCl for growth and grew at pH 6.0–11.0, with optimum growth at pH 8.0 and 0% (w/v) NaCl (Table 3). Compared with *A. towneri* DSM 14962ᵀ and *A. calcoaceticus* ATCC 23055ᵀ, the two novel strains showed a broader growth range with respect to temperature and NaCl tolerance.

**Table 3.** Comparative phenotypic characteristics and major fatty acid contents (%) of *Acinetobacter pengchengensis* and phylogenetically related species

Characteristic	LH3_13**^T^**	NS4_7	*A. towneri* DSM 14962**^T^**	*A. calcoaceticus*† ATCC 23055**^T^**
Morphology	Short rods to coccobacilli	Short rods to coccobacilli	Short rods to coccobacilli	Short rods to coccobacilli
Temperature (°C)	10–42 (30-35)	10–42 (30-35)	25–41	10–37
NaCl (%, w/v) tolerance for growth	0–5	0–5	0–2	0–2
pH range for growth	6–11	6–11	na	na
Oxygen for growth	Strictly aerobic	Strictly aerobic	Strictly aerobic	Strictly aerobic
Reaction				
Nitrate reaction	−	−	−	−
d- Mannitol assimilation	−	−	−	−
Production of:				
Lipase	−	+	+	−
Cystine arylamidase	−	−	+	−
Fatty acid				
C_12 : 0_	6.3	7.0	9.2	8.4
C_12 : 0_ 3OH	6.7	6.9	6.4	6.1
C_16 : 0_	17.0	13.8	15.9	9.7
C_18 : 1_* ω*9*c*	34.5	38.9	41.3	18.0
C_18 : 0_	1.3	1.3	1.8	0.4
Summed feature 3*	27.7	21.2	22.9	39.2

*Summed features are fatty acids that cannot be resolved reliably from another fatty acids using the chromatographic conditions chosen. The MIDI system groups these fatty acids together as one feature with a single percentage of the total. Summed feature 3, C_16 : 1_* ω7*c*/*C1_6 : 1_* ω6*c.

†Data for *A. calcoaceticus* ATCC 23055T were obtained from BacDive. For *A. towneri* DSM 14962T, morphology, growth temperature and NaCl tolerance data were obtained from BacDive. na, data not available.

As detailed in Table 3, both novel isolates were negative for nitrate reduction, glucose fermentation, aesculin hydrolysis and assimilation of ᴅ-glucose, ᴅ-mannose, *N*-acetyl-glucosamine or maltose. These biochemical features were generally consistent with those of *A. towneri* DSM 14962^T^. In the API ZYM assays, both LH3_13^T^ and NS4_7 were negative for cystine arylamidase, whereas *A. towneri* DSM 14962^T^ showed a positive reaction for this enzyme. By contrast, lipase (C14) activity was variable between the two novel strains, being negative in LH3_13^T^ and positive in NS4_7, and was, therefore, not considered a stable diagnostic feature of the novel species. Among the enzymatic activities tested, the negative cystine arylamidase reaction represented the clearest enzymatic feature distinguishing the novel strains from their closest related species. Comparison with the type species *A. calcoaceticus* ATCC 23055ᵀ also showed an overall similar biochemical profile, while several phenotypic differences were retained at the species level.

C_18 : 1_* ω9*c was identified as the predominant fatty acid in strains LH3_13^T^ and NS4_7, consistent with the genus *Acinetobacter*. The average proportion of C_18 : 1_* ω9*c in the two isolates was 36.7%, lower than that of *A. towneri* DSM 14962^T^ (41.3%) and higher than that of the type species *A. calcoaceticus* ATCC 23055ᵀ (18.0%) (Table 3). Comparison of the FAME profiles showed overall intrageneric similarity among the compared strains, with minor quantitative differences in several major fatty acids. Reverse-phase HPLC analysis of respiratory quinones revealed that the major respiratory quinones in strain LH3_13^T^ were Q9 (63.7%) and Q8 (36.3%), consistent with some other species within the genus *Acinetobacter* [48]. Polar lipids of LH3_13^T^ included diphosphatidylglycerol, phosphatidylethanolamine, phosphatidylglycerol, one unidentified lipid, one to four unidentified phospholipids, one to two unidentified aminolipids and one to five unidentified aminophospholipids (Fig. S4). Among these, PE and PG are the major polar lipid components in the *Acinetobacter* genus [16].

Analysis of the cell wall hydrolysate demonstrated the presence of meso-DAP as the diagnostic diamino acid. The molar ratio of the amino acids was determined as Ala/Glu/Gly/Asp/meso-DAP=13.95 : 8.88 : 4.38 : 3.02 : 5.48 (Figs S5 and 6). The presence of meso-DAP, together with alanine, glutamic acid and glycine, was consistent with the peptidoglycan type A1*γ*, which is a definitive characteristic of the genus *Acinetobacter* [3]. Ribose was identified as the major whole-cell sugar in strain LH3_13^T^ (Fig. S7).

## Taxonomic conclusion

Taken together, the phenotypic, biochemical, chemotaxonomic, phylogenetic and genomic data support the conclusion that strains LH3_13^T^ and NS4_7 represent a novel species within the genus *Acinetobacter*, for which the name *Acinetobacter pengchengensis* sp. nov. is proposed.

## Description of *Acinetobacter pengchengensis* sp. nov.

*Acinetobacter pengchengensis* (peng.cheng.en’sis. N.L. masc. adj. *pengchengensis,* referring to ‘Pengcheng’, an alternative name for Shenzhen, where the type strain was isolated)

Cells are Gram-stain-negative, catalase-positive, oxidase-negative, non-motile, and short rods to coccobacilli, 0.7–1.5×1.0–2.5 µm in size. Colony morphology on BHI agar after 2–3 days at 30℃ is greyish-white, smooth and circular with entire margins. Cells are rod-shaped in the exponential phase and predominantly coccobacillary in the stationary phase. Growth occurs at 10–42 °C (optimum, 30 °C), pH 6.0–11.0 (optimum, pH 8.0) and NaCl concentrations of 0–5% (w/v) (optimum, 0%). All biochemical test wells in the API 20NE strip were negative. In the API ZYM strip, positive reactions were observed for alkaline phosphatase, trypsin, *α*-chymotrypsin, *α*-galactosidase, *β*-galactosidase, *β*-glucuronidase, *α*-glucosidase, *β*-glucosidase, *N*-acetyl-*β*-glucosaminidase, *α*-mannosidase and *α*-fucosidase; negative reactions were observed for esterase (C4), esterase lipase (C8), leucine arylamidase, valine arylamidase, cystine arylamidase, acid phosphatase and naphthol-AS-BI-phosphohydrolase; the reaction for lipase (C14) was variable between strains. In the API 50CH test, positive results were observed for ᴅ-ribose, ᴅ-tagatose, aesculin iron citrate and potassium 5-ketogluconate; all other tests were negative. The major fatty acid is C_18 : 1_* ω*9*c*, and the principal respiratory quinones are Q9 and Q8. Polar lipids include diphosphatidylglycerol, phosphatidylethanolamine, phosphatidylglycerol, one unidentified lipid, 1–4 unidentified phospholipids, 1–2 unidentified aminolipids and 1–5 unidentified aminophospholipid(s). The primary cell-wall sugar component is ribose.

The type strain LH3_13^T^ (=JCM 37796^T^=GDMCC 1.4974^T^) was isolated from urban sewage in Longhua District, Shenzhen, China. The DNA G+C content of the genomic DNA of the type strain is 41.1 mol%. The GenBank accession numbers of the nearly full-length 16S rRNA gene sequences of strains LH3_13^T^ and NS4_7 are PQ035156 and PQ035159, respectively. The draft genome sequence accession numbers are JBOWQO000000000 and JBQUYF000000000, respectively. The corresponding ENA assembly accession numbers for the final hybrid genome assemblies are ERZ29227411 and ERZ29227412, respectively.

## Supplementary material

10.1099/ijsem.0.007174 Supplementary Material 1.

## Abbreviations

ANI: average nucleotide identity
BHI: brain heart infusion
CARD: Comprehensive Antibiotic Resistance Database
CCUG: Culture Collection University of Gothenburg
2D: two-dimensional
DAP: diaminopimelic acid
ENA: European Nucleotide Archive
FAMEs: fatty acid methyl esters
GDMCC: Guangdong Microbial Culture Collection Center
JCM: Japan Collection of Microorganisms
ML: maximum-likelihood
MP: maximum parsimony
NJ: neighbour-joining
R2A: Reasoner’s 2A agar
VFDB: Virulence Factor Database
