# Supplementary material for: Acinetobacter pengchengensis sp. nov., isolated from the urban wastewater of Shenzhen, Guangdong Province, China
Source: Int J Syst Evol Microbiol. 2026 Jun 17;76(6):007174. doi: 10.1099/ijsem.0.007174 (PMC13274841; doi:10.1099/ijsem.0.007174)
Supplement: Supplementary Material 1. [file ijsem-76-07174-s001.pdf]

Acinetobacter pengchengensis sp. nov. isolated from the urban wastewater of Shenzhen, Guangdong province, China

Panpan Yang[1,2], Bingchan Guo[1,2], Yinghui Li[2], Dingjie Huang[2], Xi Yang[2], Lei Wang[2], Ziqi Wu[2], Yanpeng Cheng[2*], Qinghua Hu[1*]

[1] School of Public Health, Shanxi Medical University, Taiyuan 030000, China

[2] Shenzhen Center for Disease Control and Prevention, Shenzhen 518000, China

*** Correspondence:** Qinghua Hu, School of Public Health, Shanxi Medical University, Taiyuan, China and Shenzhen Center for Disease Control and Prevention, Shenzhen, Guangdong Province, China. Email: huqinghua03@163.com; Yanpeng Cheng, Shenzhen Center for Disease Control and Prevention, Shenzhen, 518000, PR China. Email: hiwasax@foxmail.com.

Table S1. Genome assembly, annotation and quality statistics of strains LH3_13^T and NS4_7.

Genome features	LH3_13^T	NS4_7
Sequencing platform	Oxford Nanopore + Illumina HiSeq PE150	Oxford Nanopore + Illumina HiSeq PE150
Assembly method	Unicycler v0.4.7	Unicycler v0.4.7
Genome size (bp)	2,827,812	3,061,065
DNA G+C content (mol%)	41.1	41.1
No. of contigs	3	6
N50 (bp)	2,797,954	2,772,018
Total genes	2,758	2,980
Protein-coding genes (CDSs)	2,659	2,879
rRNA genes	21	21
tRNA genes	77	79
tmRNA genes	1	1
Completeness (%)	100.0	100.0
Contamination (%)	0.01	0.07
Accession no.	ERZ29227411	ERZ29227412

Footnote: The accession numbers listed in this table correspond to the ENA assembly analysis records of the final hybrid genome assemblies. The associated ENA sample accession numbers are ERS29438031 for strain LH3_13^T and ERS29438032 for strain NS4_7, and the corresponding BioProject accession numbers are PRJEB109290 and PRJEB109291, respectively.

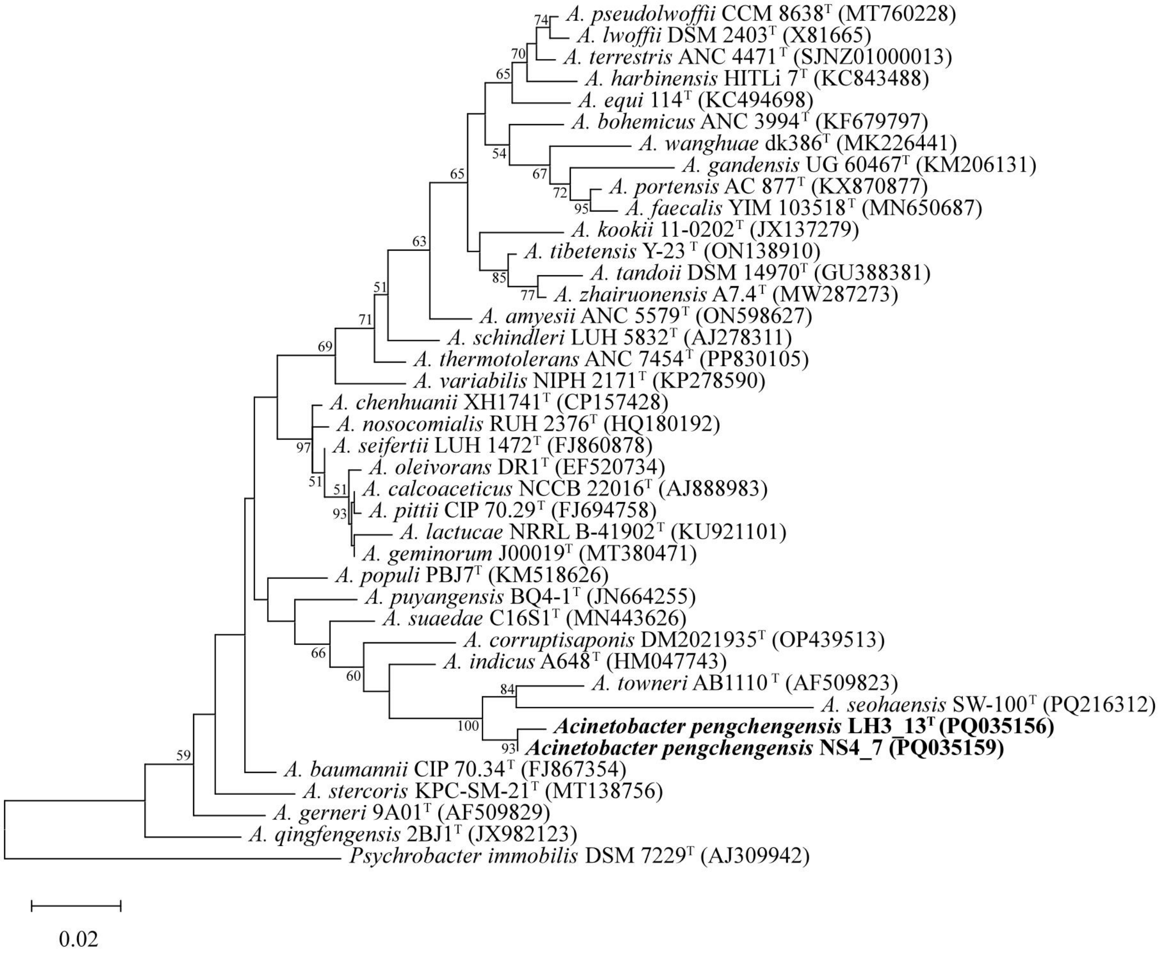

Figure S1. Phylogenetic tree created by the maximum-likelihood method based on 16S rRNA gene.

The phylogenetic tree was reconstructed with the maximum-likelihood algorithm based on 16S rRNA gene of the genus *Acinetobacter*. The sequence of *Psychrobacter immobilis* DSM 7229^{T} serves as an outgroup. Bar, 0.02 substitutions per nucleotide position.

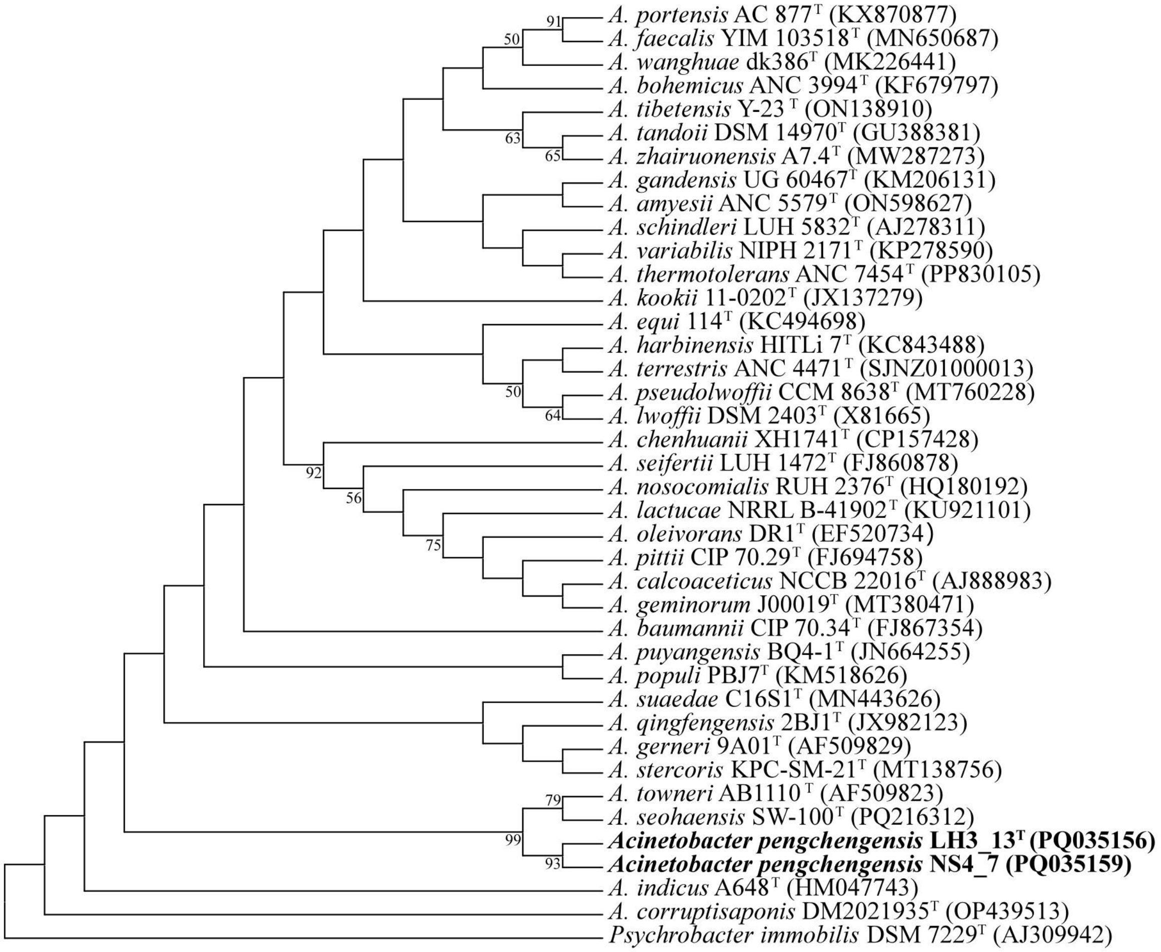

Figure S2. Phylogenetic tree created by the maximum-parsimony method based on 16S rRNA gene.

The phylogenetic tree was reconstructed with the maximum-parsimony algorithm based on 16S rRNA gene of the genus *Acinetobacter*. The sequence of *Psychrobacter immobilis* DSM 7229^{T} serves as an outgroup.

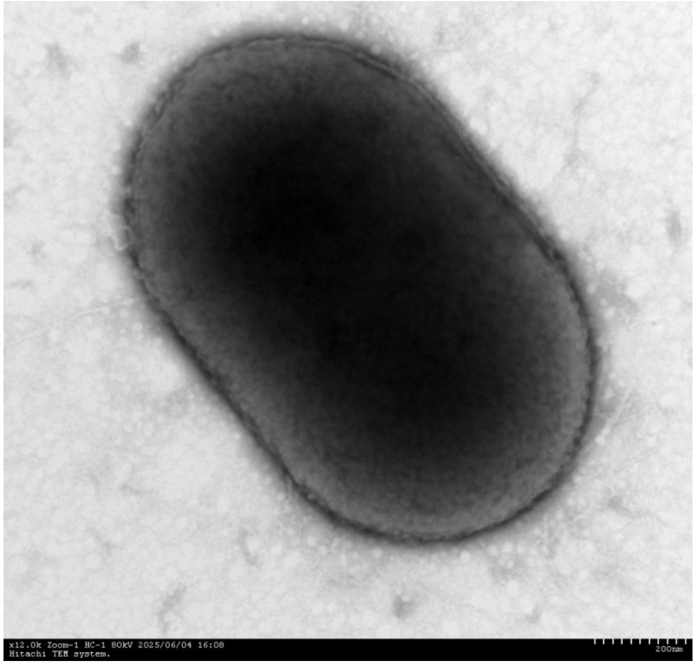

Figure S3. Transmission electron micrograph of strain LH3_13^{T} grown on BHI medium at 28°C for 2 days. Bar, 200nm.

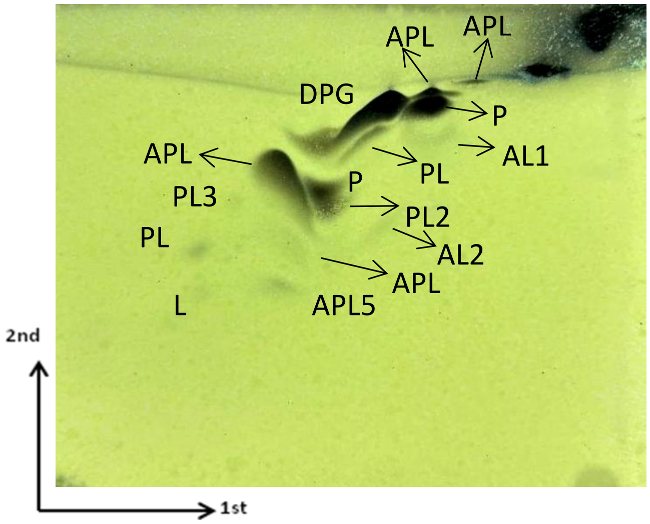

Figure S4. Two-dimensional thin-layer chromatogram of polar lipids of strain LH3_13^{T} , stained with molybdatophosphoric acid.

DPG, diphosphatidylglycerol; PE, phosphatidylethanolamine; PG, phosphatidylglycerol; PL1-4, unidentified phospholipids; AL1-2, unidentified aminolipids; L, unidentified lipid; APL1-5, unidentified aminophospholipids.

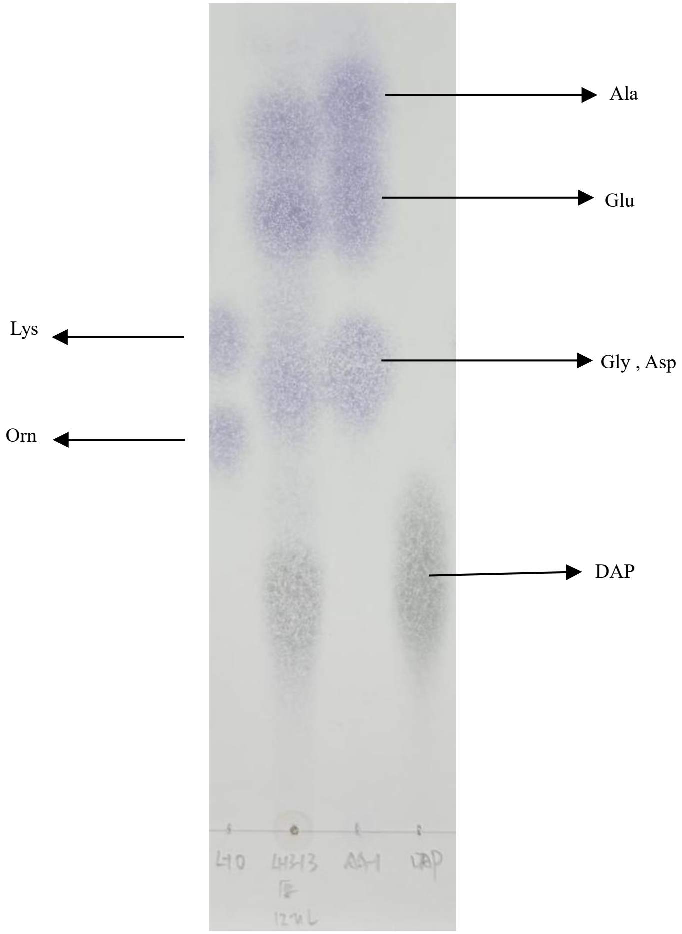

Figure S5. Thin-layer chromatographic (TLC) analysis of peptidoglycan amino acids in the cell wall of strain LH3_13^{T} （Lane 2, left to right）.

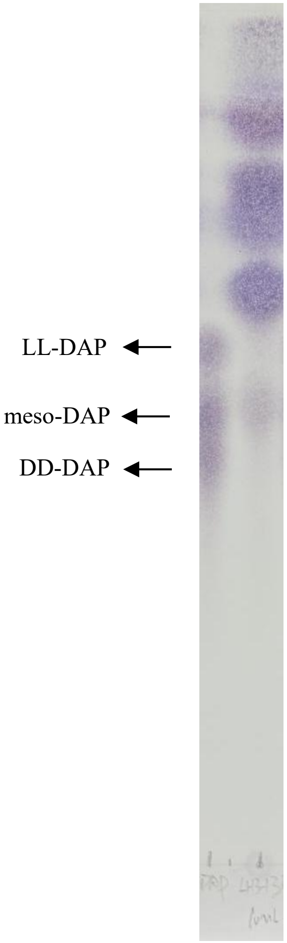

Figure S6. Thin-layer chromatographic (TLC) analysis of diaminopimelic acid (DAP) isomers.

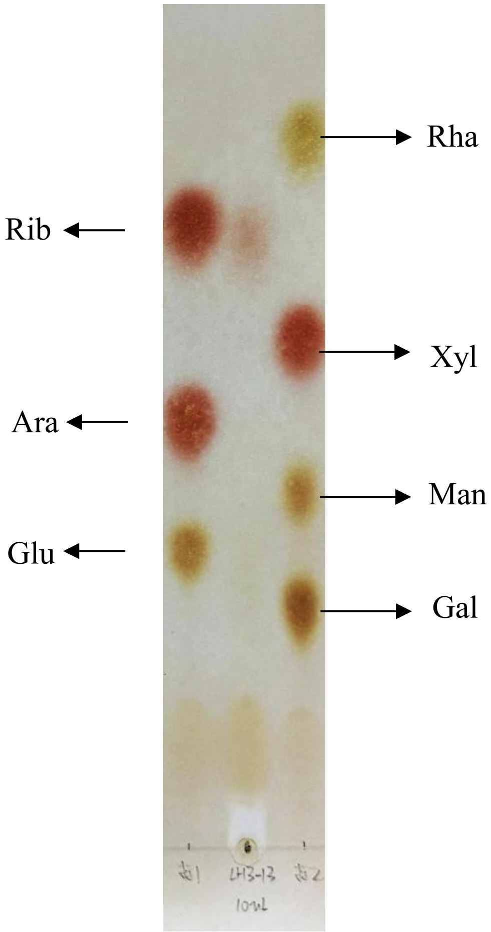

Figure S7. Chromatographic analysis of whole-cell sugars of strain LH3_13^{T}.

The positions of reference sugar standards are indicated alongside the chromatogram: Ara, arabinose; Gal, galactose; Glu, glucose; Man, mannose; Rha, rhamnose; Rib, ribose; Xyl, xylose. Ribose was identified as the major whole-cell sugar in strain LH3_13^{T}.
